# Supplementary material for: Combining machine learning with high-content imaging to infer ciprofloxacin susceptibility in isolates of Salmonella Typhimurium
Source: Nat Commun. 2024 Jun 13;15:5074. doi: 10.1038/s41467-024-49433-4 (PMC11176356; doi:10.1038/s41467-024-49433-4)
Supplement: Supplementary file 1 — Supplementary Information [file 41467_2024_49433_MOESM1_ESM.pdf]

Combining machine learning with high-content imaging to infer ciprofloxacin susceptibility in clinical isolates of *Salmonella* Typhimurium

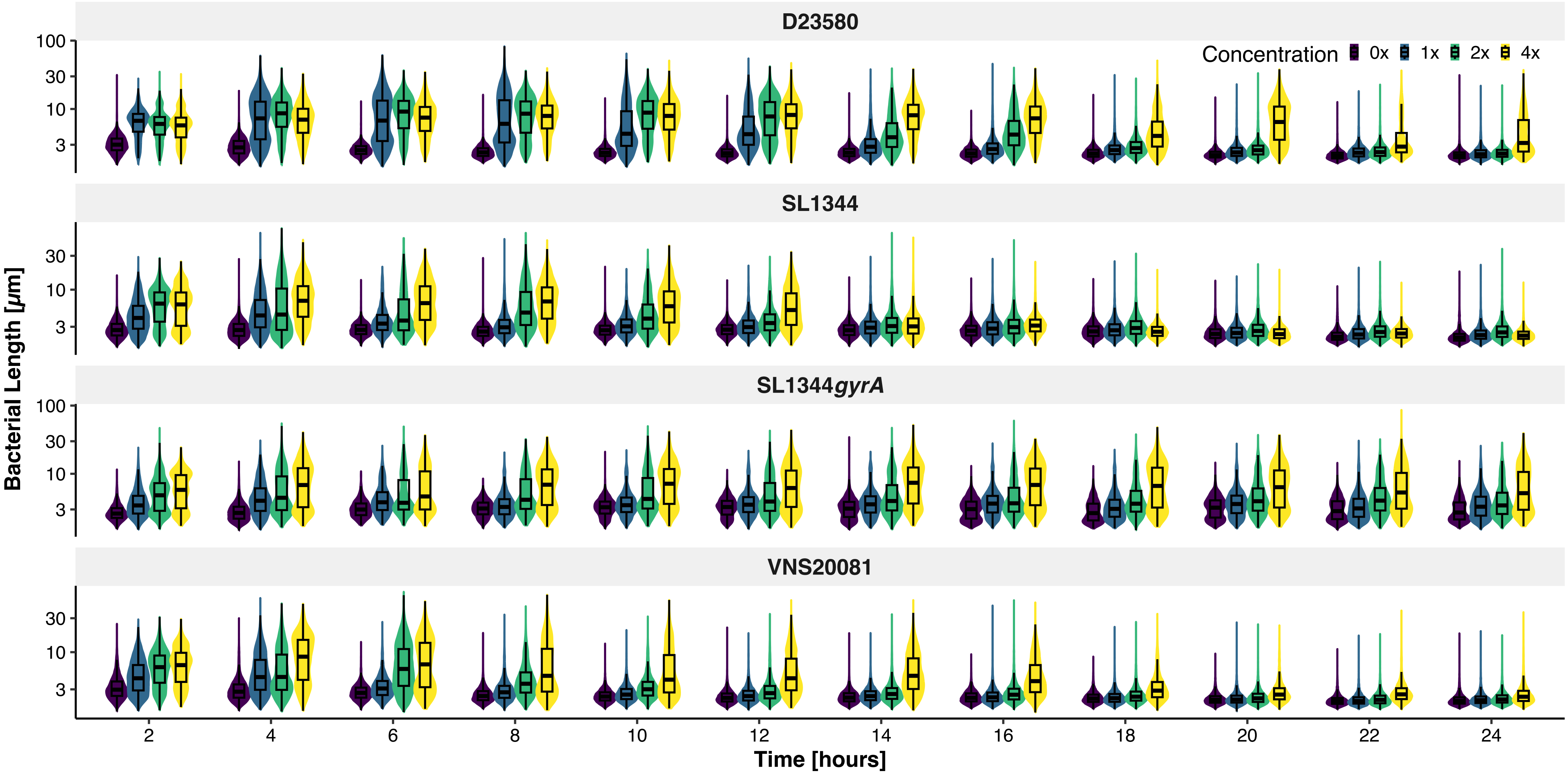

Supplementary Fig. 1. Bacterial length distribution over time from image analysis.
Violin plots showing the distribution of bacterial length in µm (y-axis) over time in hours (x-axis), according to ciprofloxacin treatment (0x, 1x, 2x, or 4x MIC). Each row of plots represents a single isolate, depicting D23580, SL1344, SL1344gyrA, and VNS20081 from top to bottom.

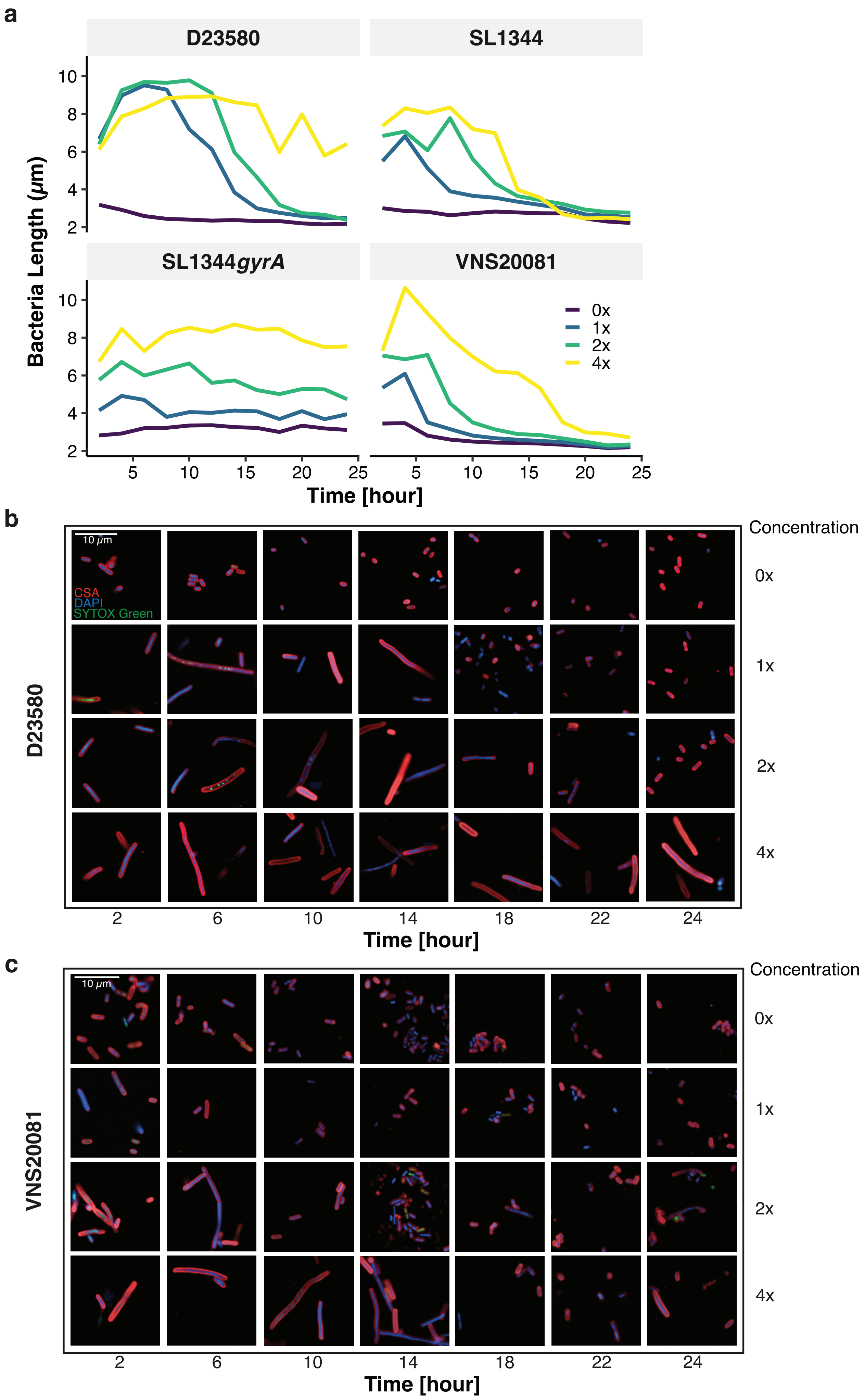

Supplementary Fig. 2. Change in bacterial length over time and ciprofloxacin exposure.
(a) Dynamics of bacterial length (vertical axis) over 24h of ciprofloxacin exposure (horizontal axis). (b) and (c) Morphologies of *S.* Typhimurium D23580 and VNS20081, respectively, at different ciprofloxacin relative concentration and exposure time. Relative concentrations (x MIC) increase from top to bottom and exposure time increases from left to right. Red, green, and blue fluorescence are from CSA, SYTOX Green, and DAPI stains, respectively.

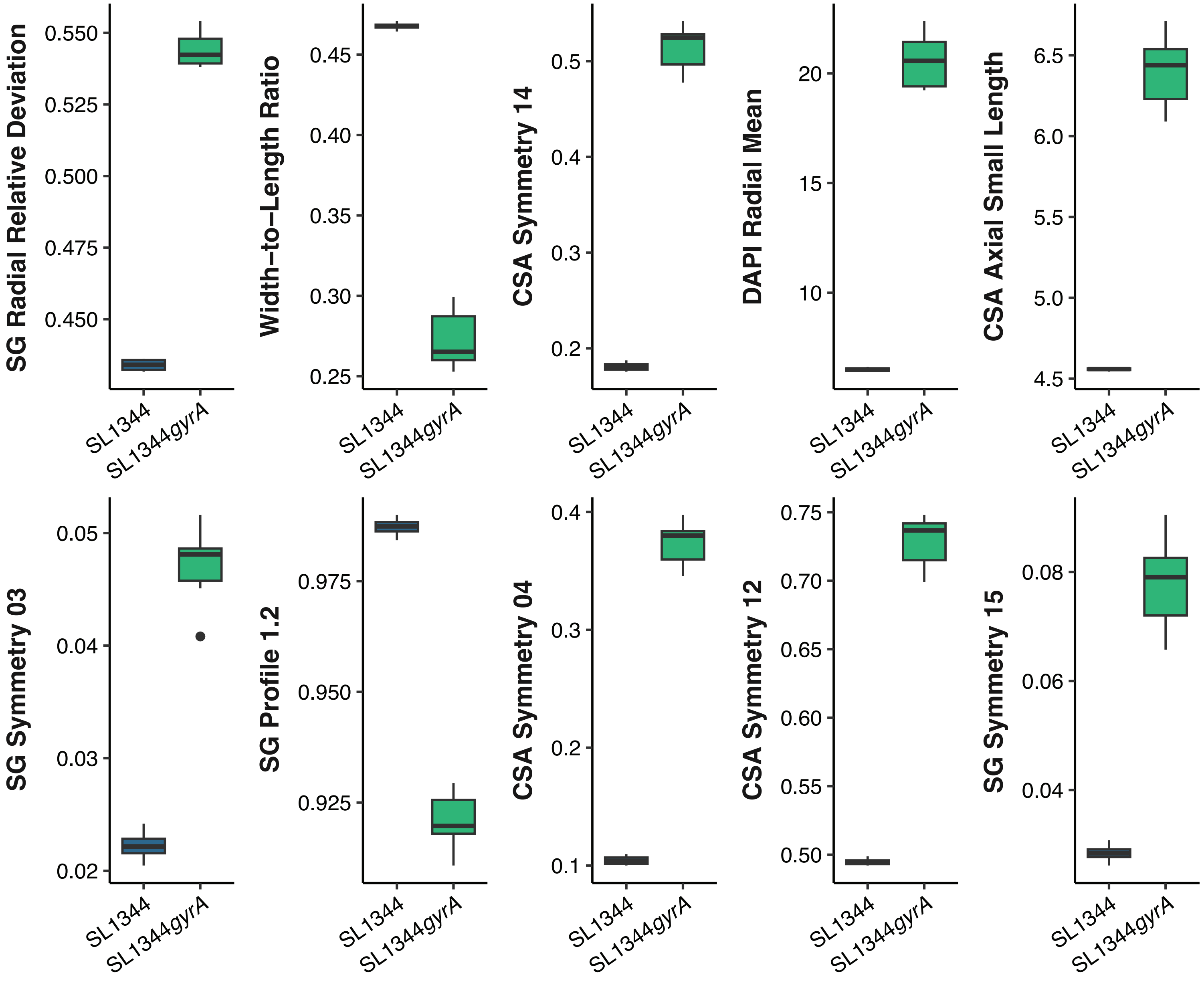

Supplementary Fig. 3. SL1344 and SL1344*gyrA* important features.
Boxplots for the ten most important features derived from the random forest. All the boxplot pairs were significantly different with p-value =0.00075 (Kruskal-Wallis test) for each pairwise comparison.

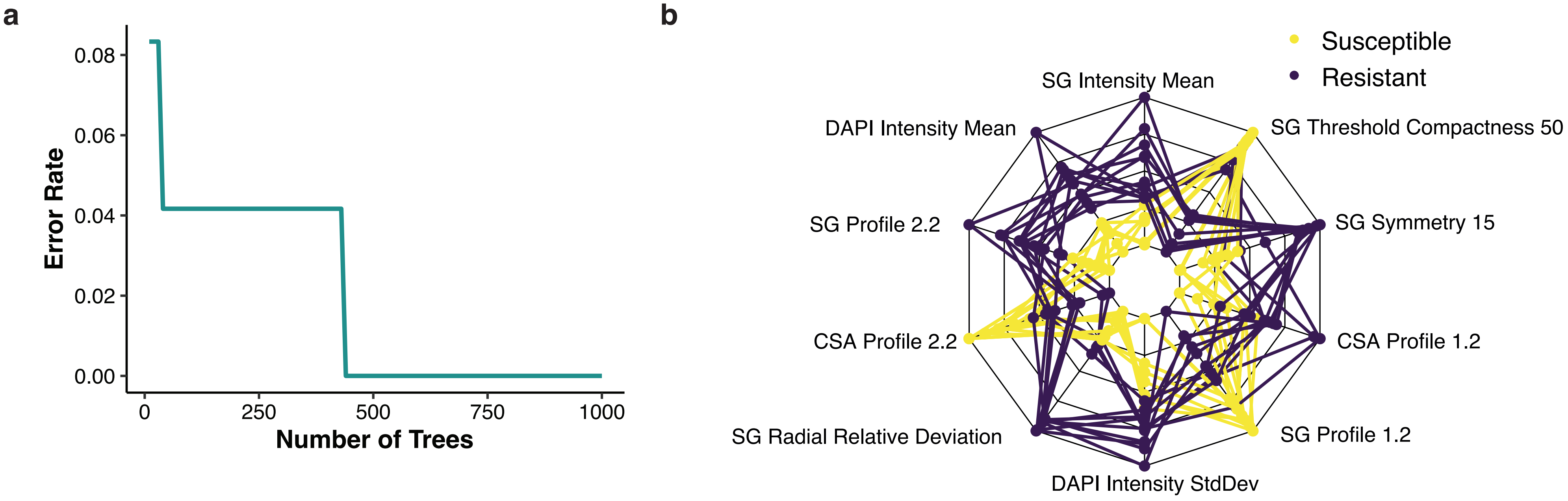

Supplementary Fig. 4. Random forest model differentiation of resistant and susceptible isolates at 0xMIC-22h.
(a) OOB error rates of random forest models trained from data at 0xMIC-22h. Horizontal axis is number of decision trees used in random forest ensembles, and vertical axis is respective OOB error rate. (b) Spider chart for the ten most important features to differentiate susceptible (yellow) and resistant (dark purple) isolates. Data was transferred to z-score, and each line is a datapoint.

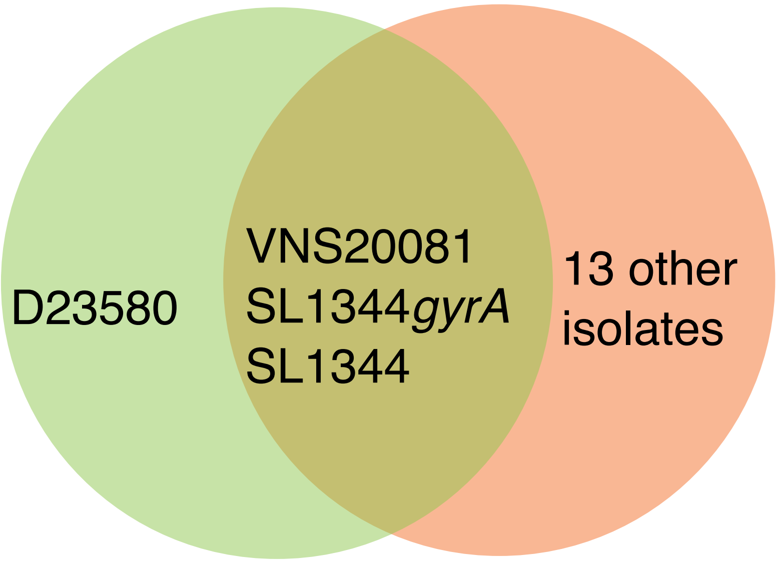

Supplementary Fig. 5. Isolates evaluated in machine learning classifier testing, training, and validation sets. Venn diagram of isolates used to train machine learning classifiers. Green circle represents the four main isolates and orange circle represents 16 isolates to test the generalization of the classifiers.

Supplementary Table 1. Evaluation of different machine learning methods to identify resistant *S.* Typhimurium on training, validation, and test sets.

	Training set						Validation set						Test set					
Method	Accuracy	Sensitivity	Specificity	Precision	F1 score	AUC	Accuracy	Sensitivity	Specificity	Precision	F1 score	AUC	Accuracy	Sensitivity	Specificity	Precision	F1 score	AUC
Naïve Bayes	0.79±0.06	0.64±0.12	0.93±0.04	0.89±0.07	0.74±0.10	0.90±0.05	0.73±0.13	0.61±0.25	0.86±0.14	0.84±0.16	0.68±0.20	0.90±0.11	0.74±0.13	0.63±0.24	0.87±0.15	0.85±0.15	0.69±0.20	0.91±0.10
KNN	0.86±0.04	0.79±0.06	0.92±0.04	0.90±0.05	0.84±0.05	0.91±0.05	0.80±0.11	0.73±0.20	0.87±0.11	0.87±0.12	0.77±0.16	0.91±0.09	0.81±0.11	0.74±0.20	0.88±0.11	0.88±0.12	0.78±0.15	0.91±0.08
SVM	0.87±0.04	0.84±0.07	0.91±0.05	0.89±0.06	0.86±0.05	0.92±0.05	0.80±0.09	0.77±0.15	0.84±0.12	0.85±0.11	0.80±0.11	0.92±0.09	0.81±0.09	0.78±0.14	0.84±0.12	0.85±0.11	0.81±0.11	0.92±0.09
Random forest	0.71±0.06	0.99±0.02	0.45±0.11	0.62±0.06	0.76±0.04	0.92±0.04	0.73±0.10	0.99±0.03	0.45±0.19	0.68±0.10	0.80±0.07	0.92±0.08	0.74±0.09	0.99±0.03	0.46±0.18	0.68±0.10	0.80±0.07	0.92±0.07
CatBoost	0.64±0.06	1.00±0.01	0.31±0.11	0.57±0.06	0.73±0.04	0.87±0.05	0.68±0.09	1.00±0.02	0.32±0.17	0.63±0.09	0.77±0.07	0.87±0.09	0.68±0.09	1.00±0.02	0.32±0.16	0.63±0.08	0.77±0.06	0.88±0.08
Neural network	0.83±0.06	0.83±0.12	0.83±0.10	0.82±0.08	0.82±0.07	0.91±0.04	0.88±0.08	0.88±0.11	0.89±0.11	0.91±0.09	0.89±0.08	0.91±0.08	0.87±0.08	0.87±0.12	0.88±0.12	0.90±0.10	0.87±0.08	0.91±0.07

Evaluation metrics are reported in mean ± standard deviation.

Supplementary Table 2. Isolates used in this study and their associated accession numbers for whole genome sequencing data.

Isolate	Accession number
SL1344	https://www.ebi.ac.uk/ena/browser/view/FQ312003
SL1344*gyrA*	https://www.ebi.ac.uk/ena/browser/view/ERS3752777
D23580	https://www.ebi.ac.uk/ena/browser/view/FN424405.1
VNS20081	https://www.ebi.ac.uk/ena/browser/view/ERS126863
VNB1779	https://www.ebi.ac.uk/ena/browser/view/ERS529943
VNB2315	https://www.ebi.ac.uk/ena/browser/view/ERS529950
gha113289	https://www.ebi.ac.uk/ena/browser/view/ERR984816
gha200597	https://www.ebi.ac.uk/ena/browser/view/ERR984817
2101	https://www.ebi.ac.uk/ena/browser/view/ERS3501145
16755_3	https://www.ebi.ac.uk/ena/browser/view/ERR2650579
5390_4	https://www.ebi.ac.uk/ena/browser/view/ERR2650639
8314_12	https://www.ebi.ac.uk/ena/browser/view/ERR2650678
8599_13	https://www.ebi.ac.uk/ena/browser/view/ERR2650685
319_8	https://www.ebi.ac.uk/ena/browser/view/ERS2313054
1304	https://www.ebi.ac.uk/ena/browser/view/ERS2313069
D23580*gyrA*	https://www.ebi.ac.uk/ena/browser/view/ERS3752774
10433_3	https://www.ebi.ac.uk/ena/browser/view/ERS3501154
