# Supplementary material for: Combining machine learning with high-content imaging to infer ciprofloxacin susceptibility in isolates of Salmonella Typhimurium
Source: Nat Commun. 2024 Jun 13;15:5074. doi: 10.1038/s41467-024-49433-4 (PMC11176356; doi:10.1038/s41467-024-49433-4)
Supplement: Supplementary file 3 — Reporting Summary [file 41467_2024_49433_MOESM3_ESM.pdf]

nature portfolio

Corresponding author(s): Stephen Baker

Last updated by author(s): May 7, 2024

Reporting Summary

Nature Portfolio wishes to improve the reproducibility of the work that we publish. This form provides structure for consistency and transparency in reporting. For further information on Nature Portfolio policies, see our Editorial Policies and the Editorial Policy Checklist.

Statistics

For all statistical analyses, confirm that the following items are present in the figure legend, table legend, main text, or Methods section.

n/a	Confirmed	
☐	☒	The exact sample size (*n*) for each experimental group/condition, given as a discrete number and unit of measurement
☒	☐	A statement on whether measurements were taken from distinct samples or whether the same sample was measured repeatedly
☐	☒	The statistical test(s) used AND whether they are one- or two-sided<br>*Only common tests should be described solely by name; describe more complex techniques in the Methods section.*
☒	☐	A description of all covariates tested
☒	☐	A description of any assumptions or corrections, such as tests of normality and adjustment for multiple comparisons
☐	☒	A full description of the statistical parameters including central tendency (e.g. means) or other basic estimates (e.g. regression coefficient) AND variation (e.g. standard deviation) or associated estimates of uncertainty (e.g. confidence intervals)
☐	☒	For null hypothesis testing, the test statistic (e.g. F, t, r) with confidence intervals, effect sizes, degrees of freedom and P value noted<br>*Give P values as exact values whenever suitable.*
☒	☐	For Bayesian analysis, information on the choice of priors and Markov chain Monte Carlo settings
☒	☐	For hierarchical and complex designs, identification of the appropriate level for tests and full reporting of outcomes
☒	☐	Estimates of effect sizes (e.g. Cohen's *d*, Pearson's *r*), indicating how they were calculated

Our web collection on statistics for biologists contains articles on many of the points above.

Software and code

Policy information about availability of computer code

Data collection	PerkinElmer Harmony High-Content Imaging and Analysis Software for the collection of confocal microscopy imaging data
Data analysis	Kraken v.1.1.1<br>Velvet v.1.2.10<br>PROKKA v1.11<br>ARIBA v.2.14.6<br>SRST2 v.0.2.0<br>SMALT v0.7.4<br>Picard v1.92<br>samtools mpileup v0.1.19<br>bcftools v0.1.19<br>Gubbins v1.4.10<br>snp-sites v.2.5.1<br>R 4.0.5<br>Matlab R2021a<br>Python 3.5<br>vegan, pheatmap, fmsb, ggplot2, ggsci, ggpubr, and randomForestSRC package on R<br>CatBoost library on Python<br>Neural Network toolbox, and Statistics and Machine Learning toolbox on Matlab

For manuscripts utilizing custom algorithms or software that are central to the research but not yet described in published literature, software must be made available to editors and reviewers. We strongly encourage code deposition in a community repository (e.g. GitHub). See the Nature Portfolio guidelines for submitting code & software for further information.

Data

Policy information about availability of data

All manuscripts must include a data availability statement. This statement should provide the following information, where applicable:

- Accession codes, unique identifiers, or web links for publicly available datasets
- A description of any restrictions on data availability
- For clinical datasets or third party data, please ensure that the statement adheres to our policy

Sequence data that support the findings of this study are available at SRA, accession IDs per isolate are available in Supplementary Data. All data generated during and/or analysed the current study are available from the corresponding author on request. Exchange of biological material should always be in agreement with the local teams.

Research involving human participants, their data, or biological material

Policy information about studies with human participants or human data. See also policy information about sex, gender (identity/presentation), and sexual orientation and race, ethnicity and racism.

Reporting on sex and gender	NA
Reporting on race, ethnicity, or other socially relevant groupings	NA
Population characteristics	NA
Recruitment	NA
Ethics oversight	NA

Note that full information on the approval of the study protocol must also be provided in the manuscript.

Field-specific reporting

Please select the one below that is the best fit for your research. If you are not sure, read the appropriate sections before making your selection.

☒ Life sciences ☐ Behavioural & social sciences ☐ Ecological, evolutionary & environmental sciences

For a reference copy of the document with all sections, see nature.com/documents/nr-reporting-summary-flat.pdf

Life sciences study design

All studies must disclose on these points even when the disclosure is negative.

Sample size	Three primary isolates of Salmonella Typhimurium and one isogenic strain were determined as representative strains for this study, based on prior investigation of these strains and their varied ciprofloxacin susceptibility. The additional 13 Salmonella Typhimurium strains used for machine learning-based analysis were determined based on diversity of ciprofloxacin susceptibility, given the strains available to the authors for experimentation.
Data exclusions	No data were excluded.
Replication	All laboratory experiments were conducted independently in triplicate using standard laboratory equipment.
Randomization	Each isolate of Salmonella Typhimurium used in experiments was tested independently of other isolates. As described above, the isolates tested were chosen based on strain availability and coverage of ciprofloxacin susceptibility.
Blinding	Blinding was not relevant to this study, as this was a descriptive study to evaluate an imaging-based methodology for determination of ciprofloxacin susceptibility in bacterial isolates of Salmonella Typhimurium.

Reporting for specific materials, systems and methods

We require information from authors about some types of materials, experimental systems and methods used in many studies. Here, indicate whether each material, system or method listed is relevant to your study. If you are not sure if a list item applies to your research, read the appropriate section before selecting a response.

Materials & experimental systems

n/a	Involved in the study
☒	☐ Antibodies
☒	☐ Eukaryotic cell lines
☒	☐ Palaeontology and archaeology
☒	☐ Animals and other organisms
☒	☐ Clinical data
☒	☐ Dual use research of concern
☒	☐ Plants

Methods

n/a	Involved in the study
☒	☐ ChIP-seq
☒	☐ Flow cytometry
☒	☐ MRI-based neuroimaging

Plants

Seed stocks	NA
Novel plant genotypes	NA
Authentication	NA
